# Microenvironmental and transcriptional determinants of the robustness of spinal cord regeneration in zebrafish

**DOI:** 10.64898/2026.09.07.749713

**Published:** 2026-09-14

**Authors:** Pierre Gillotay, Sushant Bangru, Jingwen Shen, Bethany Moore, Jianhong Ou, Ron Stewart, Kenneth D. Poss

## Abstract

Unlike mammals, adult zebrafish can regenerate spinal cord tissue after complete transection. We report here that a substantial proportion of zebrafish receiving this injury fail to regain locomotor function, and we analyzed this critically. After stratifying large cohorts of injured animals according to functional recovery, we directly compared recovered and non-recovered individuals at matched post-injury stages. We find that permanently paralyzed animals form a tissue bridge across the lesion yet exhibit impaired axonal invasion and regrowth to downstream targets. Integrative single-nucleus profiling, spatial transcriptomics, and regulatory network analyses revealed that regenerative success is characterized by the coordinated establishment of a permissive multicellular regenerative state rather than major differences in cellular composition. Furthermore, we find that regenerative failure involves persistent non-permissive regulatory programs, including extracellular matrix remodeling, impaired adaptive T-cell activation, and reduced axon growth-associated signaling. Gene regulatory network and cellular trajectory analyses identified candidate transcriptional regulators that could not be resolved through differential expression analysis alone. Functional CRISPR F _0_ screening of several of these candidates identified *hoxb5a* as a critical determinant of regenerative outcome. Stable *hoxb5a* mutants exhibited impaired swimming recovery, disrupted bridge formation, and limited axon bridging after spinal cord injury. Together, our findings reveal key differences between success and failure in a model of elevated regenerative capacity, with *hoxb5a* acting as a regulator linking multicellular tissue remodeling to axonal regeneration and functional recovery.

## Full Text Availability

The license terms selected by the author(s) for this preprint version do not permit archiving in PMC. The full text is available from the preprint server.

